# 24-Dehydrocholesterol Reductase Facilitates Cisplatin Resistance of Non-small Cell Lung Cancer via Repressing Reactive Oxygen Species/Ferroptosis Pathway

**DOI:** 10.5812/ijpr-150017

**Published:** 2024-09-17

**Authors:** Ce Qin, Jun Yuan, Rui Zhang, Li Liu, Yue-Song Ban

**Affiliations:** 1 Cangzhou Central Hospital, Hebei, China; 2 North China Petroleum General Hospital, Cangzhou, China; 3 Cangzhou People's Hospital, Cangzhou, China; 4 Huanghua People's Hospital, Hebei, China

**Keywords:** DHCR24, NSCLC, Cisplatin Resistance, Ferroptosis, PI3K/AKT/GSK3β Pathway

## Abstract

**Background:**

Non-small-cell lung cancer (NSCLC) remains a deadly malignancy worldwide. Resistance to cisplatin (DDP) is a significant obstacle that limits the therapeutic efficacy in NSCLC patients.

**Objectives:**

This study investigated the role and mechanism of 24-dehydrocholesterol reductase (DHCR24) in DDP resistance in NSCLC cells.

**Methods:**

24-dehydrocholesterol reductase levels, ferroptosis-related molecules, and proteins involved in the PI3K/AKT/GSK3β pathway were measured. The growth capacity of the cells was evaluated, and ferroptosis was assessed by measuring MDA, GSH, Fe^2+^, and ROS levels. The impact of DHCR24 on NSCLC DDP resistance was analyzed using a tumor xenograft assay in vivo. Ki-67 and DHCR24 expression in tumors were evaluated through immunohistochemical staining.

**Results:**

24-dehydrocholesterol reductase expression was elevated in DDP-resistant cells, indicating a poorer prognosis for NSCLC patients. Down-regulation of DHCR24 inhibited the growth of DDP-resistant cells and induced ferroptosis. Inhibition of DHCR24 led to the inactivation of the PI3K/AKT/GSK3β pathway and subsequent induction of ferroptosis. Inhibition of ferroptosis or activation of the PI3K/AKT/GSK3β pathway counteracted the increased DDP sensitivity induced by DHCR24 knockdown in NSCLC cells. Additionally, DHCR24 deficiency improved NSCLC DDP resistance in vivo.

**Conclusions:**

24-dehydrocholesterol reductase contributes to DDP resistance in NSCLC cells by suppressing ferroptosis through the activation of the PI3K/AKT/GSK3β pathway.

## 1. Background

As a systemic therapy, chemotherapy has been recognized as the primary treatment method for non-small-cell lung cancer (NSCLC) patients, significantly improving the 5-year survival rate by about 5 - 15% (1). Cisplatin (DDP) is the first-line chemotherapy drug for NSCLC and is frequently used in clinical settings (2). However, resistance to DDP can greatly reduce treatment efficacy, leading to recurrence and metastasis (3). Advancements in molecular biology offer innovative opportunities for identifying targeted therapies against drug-resistant NSCLC. Therefore, it is urgent to uncover the mechanisms underlying DDP resistance to increase NSCLC patients' sensitivity to cisplatin therapy and ultimately improve outcomes.

Ferroptosis is a new type of cell death that occurs in an iron-dependent manner, characterized by glutathione (GSH) exhaustion and reactive oxygen species (ROS) overproduction (4). Glutathione peroxidase 4 (GPX4) is a crucial suppressor of ferroptosis, and its activity can be modulated by GSH (5). Increasing evidence suggests that ferroptosis is closely associated with tumor growth and chemoresistance (6). Ferroptosis induction has been reported as an effective strategy to overcome cisplatin resistance in NSCLC cells (7). Despite extensive basic research, the regulatory mechanisms of ferroptosis underlying NSCLC cisplatin resistance remain unclear.

24-dehydrocholesterol reductase (DHCR24) is an important regulatory enzyme in cholesterol synthesis and has been documented as a ROS scavenger in colorectal cancer (8). DHCR24 plays a crucial role in tumor development and is also identified as a biomarker for tumor diagnosis (9). In HepG2 cells, repressing DHCR24 activity has been shown to increase the sensitivity of pancreatic cancer cells to chemotherapy (10, 11). A recent study reported that high expression of DHCR24 is associated with poorer outcomes in lung adenocarcinoma (12). Notably, DHCR24 was found to be up-regulated in DDP-resistant NSCLC cells (13). However, the role and mechanism of DHCR24 in the context of DDP resistance during NSCLC development have not yet been investigated.

## 2. Objectives

The biological function of DHCR24 in DDP resistance was investigated using DDP-resistant NSCLC cells and nude mice.

## 3. Methods

### 3.1. Clinical Samples

Thirty paired NSCLC and para-carcinoma tissues were obtained during surgical removal at Cangzhou Central Hospital. The NSCLC tissues were divided into a DDP-resistant group (n = 17) and a DDP-sensitive group (n = 13). All NSCLC patients enrolled in this study provided informed consent.

### 3.2. Cell Culture, Establishment of DDP-Resistant Cells, and Treatments

A549 and NCI-H1975 cells were cultured in F-12K and RPMI-1640 (Gibco, USA), respectively, with 10% FBS (Gibco). To establish DDP-resistant cells, NSCLC cells were exposed to increasing concentrations of DDP according to a previous study (14). Briefly, A549 and NCI-H1975 cells were treated with 0.5 μg/mL DDP (MCE, USA) for 3 days, followed by a 72-hour recovery period. Subsequently, increasing doses of DDP (1, 2, 4, 8, and 10 μg/mL) were administered. After three cycles, DDP-resistant cells were successfully established and named A549/DDP and NCI-H1975/DDP.

To inhibit ferroptosis, A549/DDP and NCI-H1975/DDP cells were treated with 10 μM Ferrostatin-1 (15), a ferroptosis inhibitor, for 24 hours. IGF-1 (100 ng/mL, MCE) (16) was administered for 24 hours to activate the PI3K/AKT pathway.

### 3.3. Cell Transfection

A549/DDP and NCI-H1975/DDP cells were transfected with sh-DHCR24 or sh-NC (GenePharma, Shanghai, China) using Lipofectamine 2000 (Thermo Fisher, USA). The stably transfected cells were selected by treatment with decreasing doses of Neomycin (ranging from 800 to 200 μg/mL) for two weeks.

### 3.4. Quantitative Real time Polymerase Chain Reaction (RT-qPCR)

Total RNA was isolated from A549/DDP and NCI-H1975/DDP cells using TRIzol (Thermo Fisher). Reverse transcription was performed with RT Master Mix for qPCR II (MCE). The mRNA expression of DHCR24 was measured using SYBR Green Premix Ex Taq ROX Plus (Takara, Japan). The relative level of mRNA was calculated using the 2^−ΔΔCT^ method. The primer sequences were as follows: DHCR24, forward: ATGGCAGCTTTGTGCGATG, reverse: ACGCAGCTTGACGTACTTCT; β-Actin, forward: TTGCGTTACACCCTTTCTTG, reverse: CACCTTCACCGTTCCAGTTT.

### 3.5. Western Blotting

DDP-resistant NSCLC cells and tumors were harvested and lysed with RIPA buffer (Beyotime, Haimen, China) to obtain total protein samples, which were then quantified using the BCA protein quantification kit (Yeasen, Shanghai, China). Protein samples (40 μg) were loaded onto SDS-PAGE gels and transferred to PVDF membranes. After blocking with 5% skim milk, the blots were incubated with primary antibodies against DHCR24 (#2033, CST, USA), GPX4 (A21440, ABclonal, Wuhan, China), SLC7A11 (A2413, ABclonal), ACSL4 (A20414, ABclonal), p-PI3K (AP0427, ABclonal), PI3K (A22487, ABclonal), p-AKT (AP1453, ABclonal), AKT (A18120, ABclonal), p-GSK3β (AP0261, ABclonal), GSK3β (A6164, ABclonal), and GAPDH (A19056, ABclonal) overnight at 4°C. This was followed by incubation with a secondary antibody (AS014, ABclonal). Protein bands were developed using ECL Western Blotting Substrate (Solarbio, Beijing, China).

### 3.6. Cell Counting Kit-8 (CCK-8)

Three thousand A549/DDP and NCI-H1975/DDP cells in 96-well plates were treated with 10 µL of CCK8 (Beyotime) for 2 hours at 37°C. Subsequently, the optical density at 450 nm (OD450) was measured using a microplate reader (Bio-Tek, USA).

### 3.7. Colony Formation

One thousand A549/DDP and NCI-H1975/DDP cells were cultured in 6-well plates for two weeks. After fixation with 4% paraformaldehyde and staining with crystal violet, the colonies were counted.

### 3.8. Detection of MDA, GSH, Fe2+, ROS Levels

The MDA, GSH, Fe²⁺, and ROS levels were determined using commercial assay kits provided by Solarbio, following the protocols for each kit.

### 3.9. Animal Model

Male, 4-week-old BALB/c nude mice (SLAC Laboratory, Shanghai, China) were divided into four groups (n = 6 per group): Control, DDP, DDP + sh-NC, and DDP + sh-DHCR24. A549/DDP cells (3 × 10^6^) were subcutaneously injected into the left flank of the mice. When the tumor diameter reached approximately 5 mm, the mice were administered DDP (intraperitoneal injection, 4 mg/kg) every 3 days for a total of 6 injections (17). Tumor volume was calculated using the formula: Volume = 1/2 × width × length². All mice were euthanized, and tumors were collected. The animal experiments were conducted in accordance with the ethics approval from the Committee on the Ethics of Cangzhou Central Hospital (ethical code: 2022-187-03(z)).

### 3.10. Immunohistochemical Staining

The xenografts were fixed in 4% paraformaldehyde, dehydrated, embedded in paraffin, and cut into 4-μm thick sections. After deparaffinization, the sections underwent antigen retrieval. They were then probed overnight at 4°C with Ki-67 (#34330, 1:200, CST) or DHCR24 (#2033, 1:100, CST), followed by incubation with a secondary antibody (AS014, 1:2000, ABclonal) and staining with 3,3′-diaminobenzidine. After washing, the sections were observed under a light microscope (Olympus, Japan).

### 3.11. Statistical Analysis

Results are presented as mean ± standard deviation (SD). Comparisons between two groups were performed using Student’s *t*-test, and comparisons among multiple groups were conducted using one-way ANOVA, all analyzed with GraphPad Prism 6.0. A P-value of < 0.05 was considered statistically significant.

## 4. Results

### 4.1. DHCR24 Was Highly Expressed During DDP Resistance

**Figure 1.**
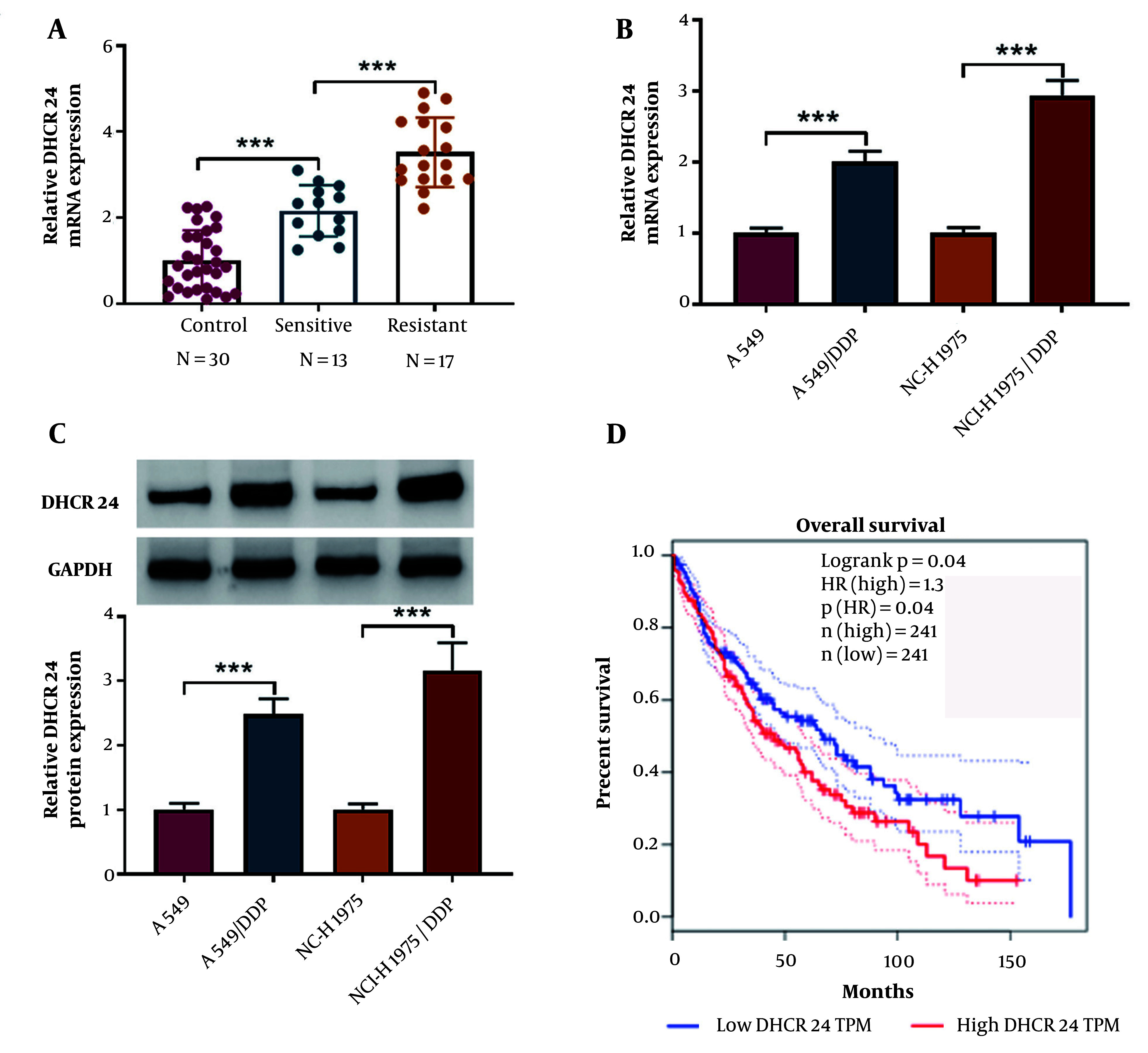
The DHCR24 was up-regulated in DDP-resistant NSCLC samples and cells. A, quantitative real time polymerase chain reaction (RT-qPCR) analysis of DHCR24 mRNA level in DDP-resistant and sensitive NSCLC tissues and para-carcinoma tissues; B and C, DHCR24 mRNA and protein expression in DDP-resistant NSCLC cells and parental cells was assessed by RT-qPCR and Western blotting; D, GEPIA database analyzed the correlation between DHCR24 expression and survival of NSCLC patients. *** P < 0.001.

We evaluated the differential levels of DHCR24 in para-carcinoma, DDP-resistant, and DDP-sensitive NSCLC tissues. Quantitative real time polymerase chain reaction (RT-qPCR) analysis showed that DHCR24 mRNA levels were higher in the DDP-sensitive group compared to the control group, with even higher levels observed in the DDP-resistant group (Figure 1A). Additionally, DHCR24 mRNA levels were elevated in DDP-resistant cells (Figure 1B). Consistently, the protein levels of DHCR24 were significantly higher in DDP-resistant NSCLC cells (Figure 1C). Furthermore, the Gepia database (http://gepia.cancer-pku.cn/detail.php) indicated that NSCLC patients with high DHCR24 expression had a lower survival rate compared to those with low DHCR24 expression (Figure 1D). Collectively, these findings suggest that DHCR24 is highly expressed in DDP resistance and is associated with poor outcomes in NSCLC.

### 4.2. DHCR24 Down-Regulation Raised DDP Sensitivity

**Figure 2.**
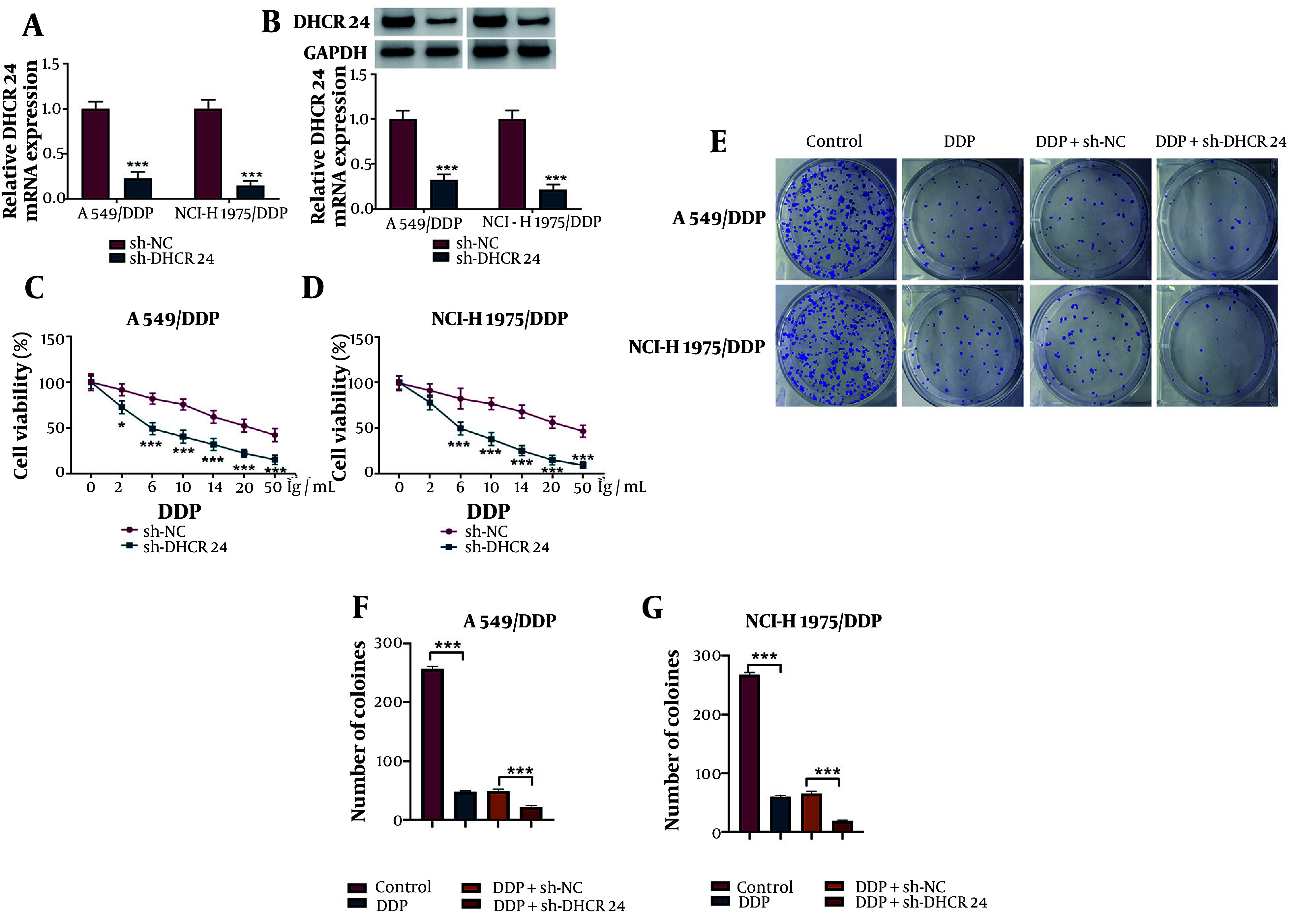
The DDP sensitivity was elevated in DHCR24-silenced NSCLC cells. A549/DDP and NCI-H1975/DDP cells were transfected with sh-DHCR24 or sh-NC. A and B, quantitative real time polymerase chain reaction (RT-qPCR) and Western blotting determined DHCR24 mRNA and protein levels; C and D, after exposure to various concentrations of DDP, cell viability was assessed by CCK-8; E-G, the growth of NSCLC cells was evaluated by colony formation assay. * P < 0.05, *** P < 0.001.

The silencing efficiency of sh-DHCR24 was validated (Figure 2A, B). Subsequently, A549/DDP and NCI-H1975/DDP cells were treated with various concentrations of DDP (0, 2, 6, 10, 14, 20, or 50 μg/mL) following DHCR24 knockdown. Cell viability was reduced in a dose-dependent manner by DDP, with the reduction further enhanced by DHCR24 silencing (Figure 2C, D). Additionally, DHCR24 knockdown further decreased the number of colonies formed in response to DDP (Figure 2E, G). These findings support the conclusion that DHCR24 silencing enhances DDP sensitivity in NSCLC cells.

### 4.3. DHCR24 Deficiency Induced Ferroptosis to Enhance DDP Sensitivity of NSCLC Cells

**Figure 3.**
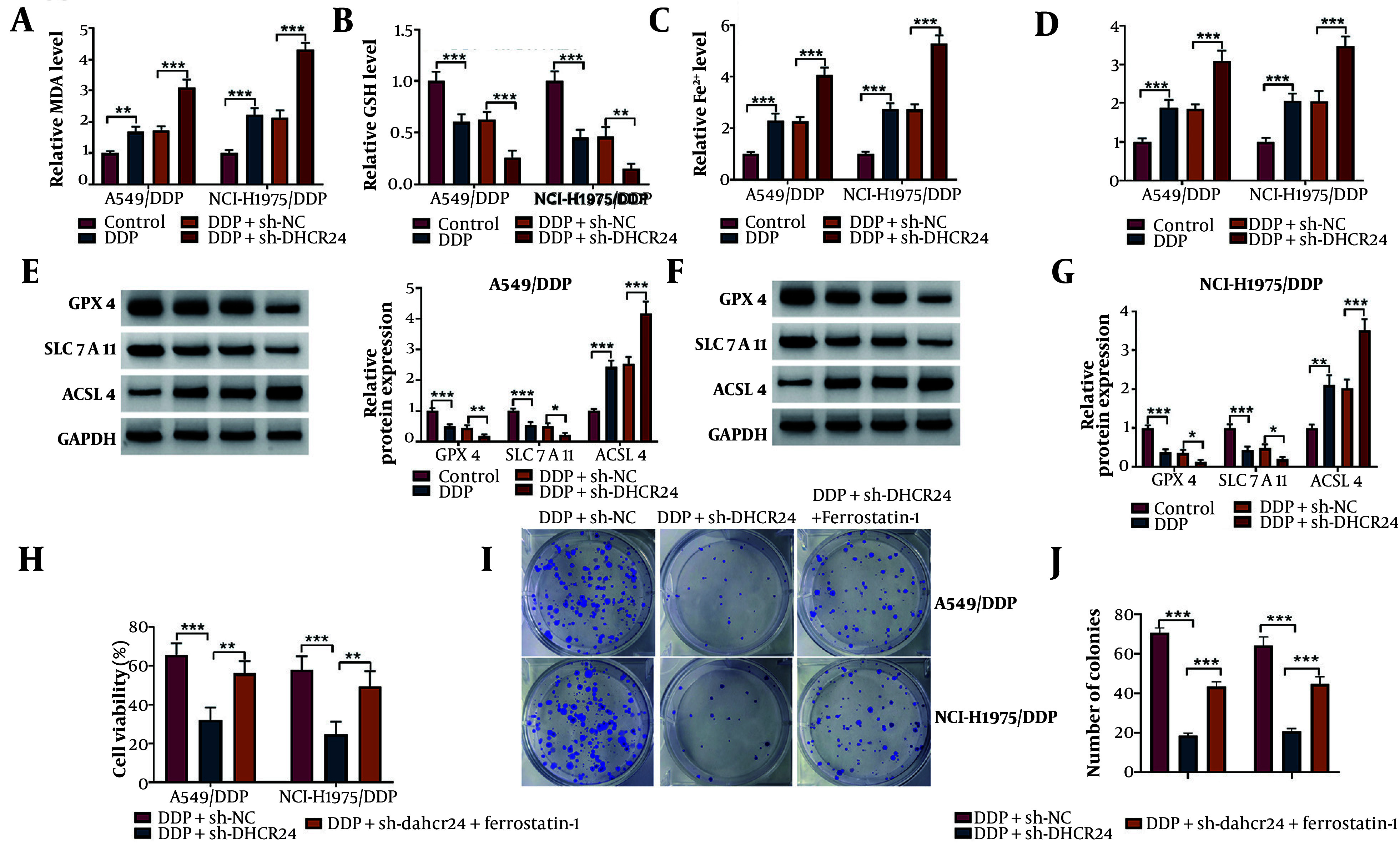
The DHCR24 knockdown enhanced DDP sensitivity of NSCLC cells via inducing ferroptosis. Sh-DHCR24 or Sh-NC-transfected A549/DDP and NCI-H1975/DDP cells were treated with 10 μg/mL DDP. A-D, the levels of MDA, GSH, Fe^2+^, and ROS levels were detected by commercial kits. E and F, the protein abundance of GPX4, LC7A11, and ACSL4 was measured by Western blotting. Sh-DHCR24-transfected A549/DDP and NCI-H1975/DDP cells were treated with 10 μM Ferrostatin-1 in the presence of DDP; G, cell viability of DDP-resistant NSCLC cells was evaluated by CCK-8; H, the growth ability was analyzed by colony formation assay. * P < 0.05, ** P < 0.01, *** P < 0.001.

Ferroptosis has been identified as a novel type of cell death that can overcome DDP resistance in NSCLC cells (18). Thus, we further explored the role of DHCR24 in regulating ferroptosis in DDP-resistant NSCLC cells. As expected, we observed an accumulation of lipid peroxidation products (MDA), Fe^2+^, and ROS, along with a depletion of GSH in DDP-treated DDP-resistant cells, which was further exacerbated by DHCR24 deficiency (Figure 3A-D). Additionally, DHCR24 depletion further decreased GPX4 and SLC7A11 levels, while increasing ACSL4 levels in A549/DDP and NCI-H1975/DDP cells (Figure 3E, F). To determine whether DHCR24 affected DDP sensitivity in NSCLC cells through modulation of ferroptosis, sh-DHCR24-transfected cells were co-treated with Ferrostatin-1, a ferroptosis inhibitor. The reduced cell viability caused by DHCR24 downregulation was partially restored by Ferrostatin-1 co-treatment (Figure 3G). Furthermore, the reduction in the number of colonies in DHCR24-deficient cells was significantly reversed by Ferrostatin-1 administration upon DDP exposure (Figure 3H). These observations suggest that DHCR24 knockdown sensitizes NSCLC cells to DDP by inducing ferroptosis.

### 4.4. DHCR24 Knockdown Triggered Ferroptosis via Inactivation of PI3K/AKT/GSK3β Pathway

**Figure 4.**
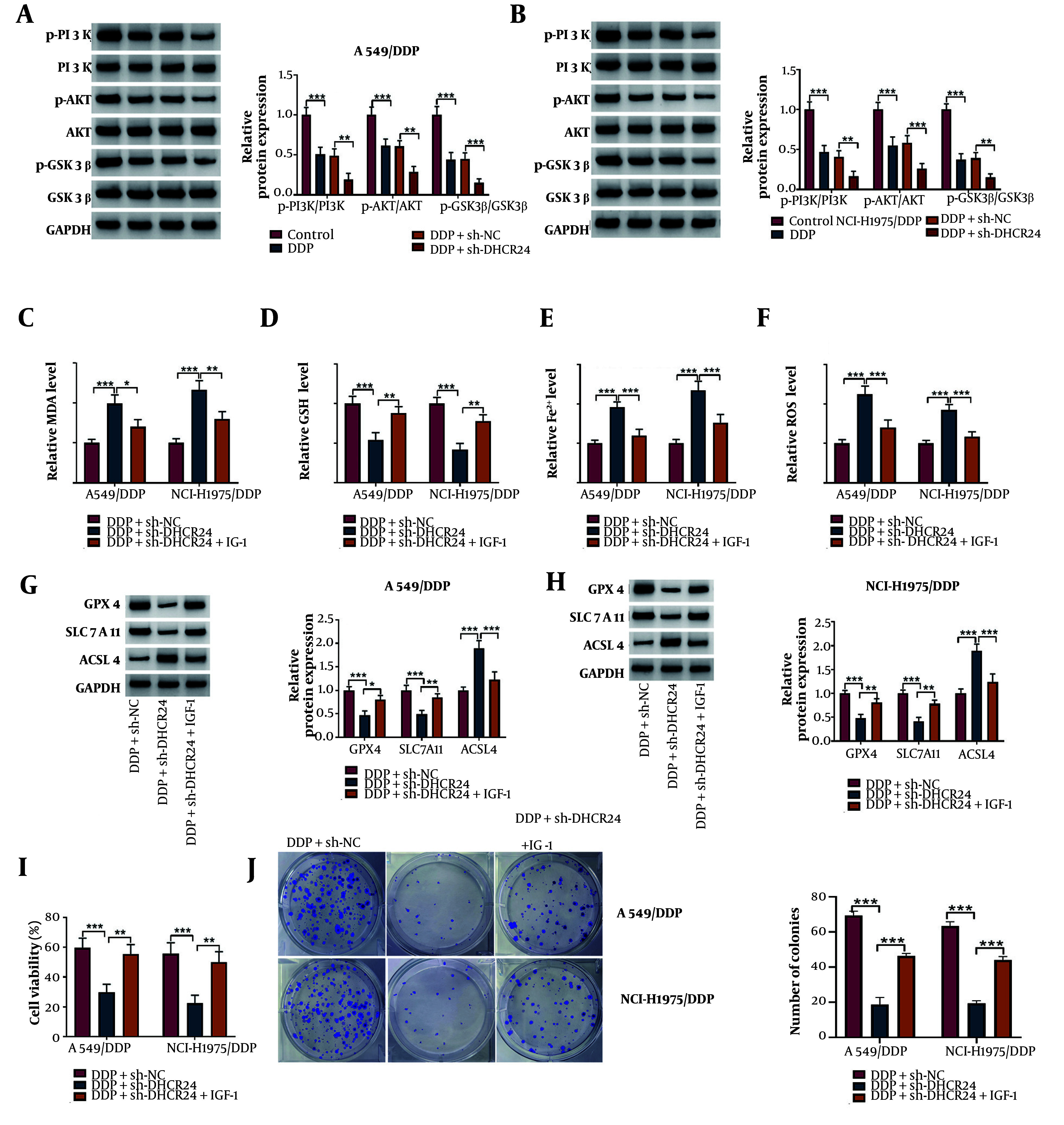
The DHCR24 down-regulation triggered ferroptosis by inactivating PI3K/AKT/GSK3β pathway. A549/DDP and NCI-H1975/DDP cells were transfected with sh-DHCR24 or Sh-NC together with treatment with 10 μg/mL DDP. A and B, the protein levels of p-PI3K, PI3K, p-AKT, AKT, p-GSK3β, and GSK3β were detected by Western blotting; C-F, MDA, GSH, Fe^2+^, and ROS levels were assessed by commercial kits. Sh-DHCR24-transfected DDP-resistant NSCLC cells were co-treated with 100 ng/mL IGF-1 upon DDP exposure. G and H, the protein abundance of GPX4, LC7A11, and ACSL4 was determined by Western blotting; I and J, the growth of cells was determined by CCK-8 and colony formation assay. * P < 0.05, ** P < 0.01, *** P < 0.001.

To further investigate the upstream regulatory mechanism of DHCR24 in ferroptosis, we focused on the PI3K/AKT/GSK3β pathway. Western blotting results showed that the levels of p-PI3K, p-AKT, and p-GSK3β proteins were significantly increased in DDP-stimulated NSCLC cells, and DHCR24 depletion further intensified these changes (Figure 4A,  B). Additionally, DHCR24-silenced cells were treated with IGF-1 to activate the PI3K/AKT/GSK3β pathway. We observed that IGF-1 treatment counteracted the elevated MDA, Fe^2+^, and ROS levels, as well as the decreased GSH content induced by sh-DHCR24 (Figure 4C-F). Moreover, IGF-1 co-treatment reversed the reductions in GPX4 and SLC7A11 levels and the increase in ACSL4 levels in DHCR24-depleted NSCLC cells (Figure 4G,H). Furthermore, the reduced cell viability and colony-forming capacity of sh-DHCR24-transfected NSCLC cells were significantly improved following IGF-1 administration (Figure 4I, J). Taken together, these results suggest that IGF-1-induced activation of the PI3K/AKT/GSK3β pathway plays a role in the ferroptosis triggered by DHCR24 knockdown in NSCLC cells.

### 4.5. DHCR24 Depletion Restrained Xenograft Growth and DDP Resistance in Vivo

**Figure 5.**
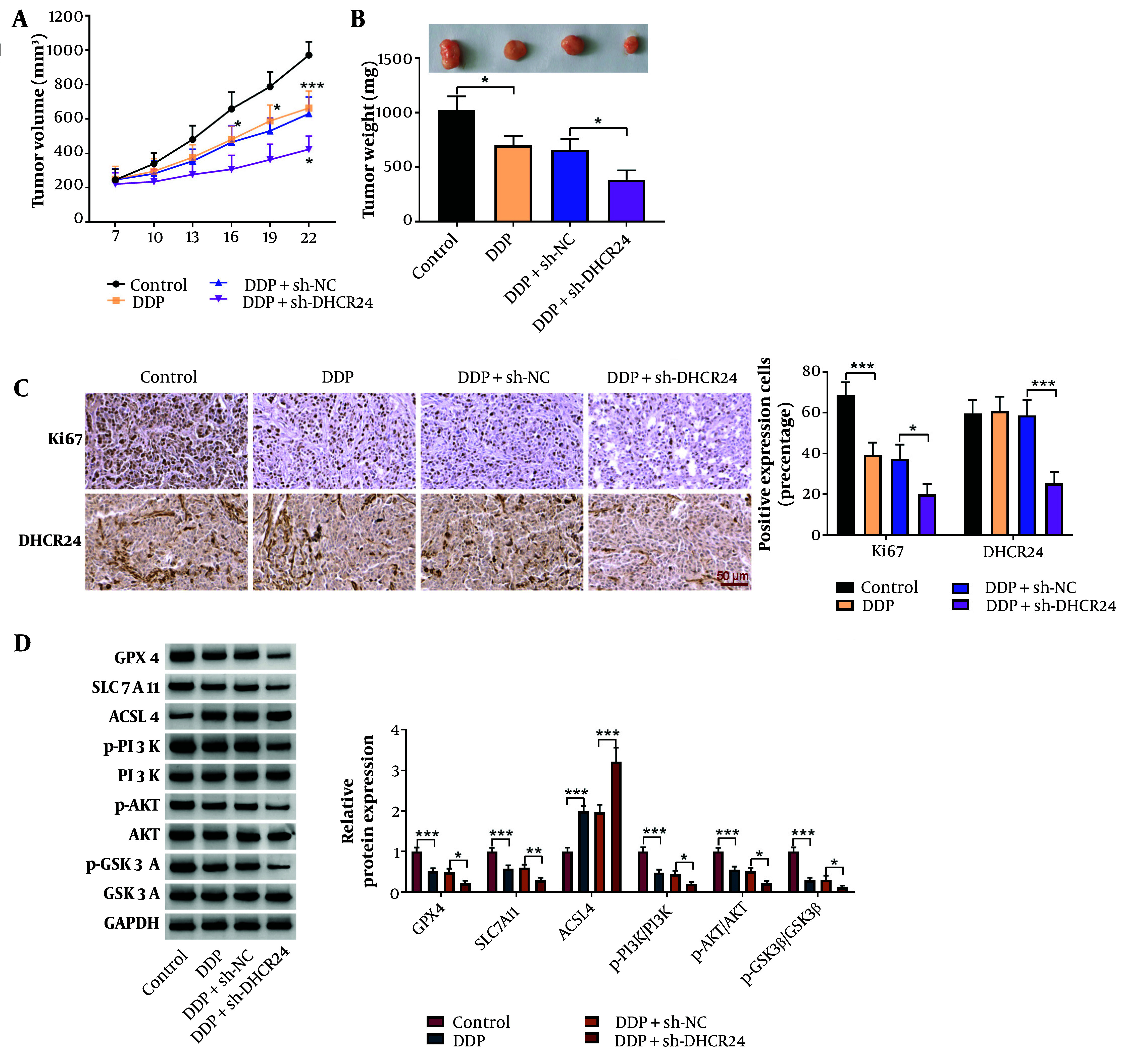
The DHCR24 silencing delayed xenograft growth and enhanced DDP sensitivity of NSCLC cells in vivo. The A, tumor volume; and B, weight were detected in different groups; C, Ki67 and DHCR24 expression in tumors was determined by immunohistochemical staining (magnification, x400); D, Western blotting analysis of GPX4, LC7A11, ACSL4, p-PI3K, PI3K, p-AKT, AKT, p-GSK3β, and GSK3β protein levels in tumor tissues. * P < 0.05, ** P < 0.01, *** P < 0.001.

Finally, we functionally verified the in vitro results in tumor-bearing mice in vivo. DHCR24 deficiency effectively increased sensitivity to DDP, as indicated by reduced tumor volume and weight (Figure 5A, B). Ki-67 expression in tumors was significantly reduced by DDP treatment, while DHCR24 expression remained unaffected (Figure 5C). However, DHCR24 silencing notably decreased both Ki-67 and DHCR24 expression in tumor tissues upon DDP treatment (Figure 5C). Furthermore, DDP treatment downregulated GPX4, SLC7A11, p-PI3K, p-AKT, and p-GSK3β protein levels while upregulating ACSL4 in tumors, with these effects further amplified by DHCR24 ablation (Figure 5D). In summary, our in vivo evidence demonstrates that DHCR24 silencing mitigates DDP resistance by inducing ferroptosis through the inactivation of the PI3K/AKT/GSK3β pathway.

## 5. Discussion

Due to its affordability and effectiveness, DDP has been considered the cornerstone of treatment for NSCLC (19). However, the development of DDP resistance has led to therapeutic failure and poor prognosis for NSCLC patients (20). The unclear molecular mechanisms underlying DDP resistance remain a major barrier to achieving effective treatment for NSCLC (21). Therefore, uncovering the molecular mechanisms of DDP resistance is crucial for overcoming it and improving treatment outcomes. DHCR24, a key enzyme in cholesterol synthesis, has been documented to affect various cellular functions, including cell proliferation, oxidative stress response, and inflammation (22). Additionally, a close association has been reported between DHCR24 upregulation and malignant behaviors in cancer cells (23). High expression of DHCR24 has also been validated in DDP-resistant NSCLC cells (13). Consistent with previous studies, we observed elevated levels of DHCR24 in DDP-resistant NSCLC cells. A study by Tian et al. revealed that silencing DHCR24 enhanced DDP-induced damage in cochlear hair cells (24). In line with this, our study provides the first evidence that DHCR24 deficiency effectively sensitizes NSCLC cells to DDP treatment.

Ferroptosis has been shown to play a role in regulating DDP resistance in cancer cells (25). Additionally, d-borneol treatment has increased NSCLC cell sensitivity to DDP by inducing ferroptosis, thus enhancing anticancer efficacy (26). DHCR24 has been identified as protective against oxidative stress-induced injury in hepatocytes (27). Another study reported that DHCR24 inhibited excessive ROS production in pancreatic β cells induced by endoplasmic reticulum stress (28). Our study aligns with these findings, showing that DHCR24 depletion induces ferroptosis, as evidenced by decreased levels of GSH, GPX4, and SLC7A11, and increased levels of MDA, ROS, and ACSL4. Furthermore, the increased DDP sensitivity of NSCLC cells induced by DHCR24 knockdown was diminished by a ferroptosis inhibitor. These findings collectively reveal that DHCR24 inhibition reduces DDP resistance in NSCLC by triggering ferroptosis.

It has been suggested that various pathways, such as the PI3K/AKT signaling pathway, may be involved in the modulation of ferroptosis (29). GSK3β is recognized as one of the downstream effectors of the PI3K/AKT pathway (30). Yao et al. found that DHCR24 mitigated H2O2-induced oxidative damage in A549 cells by activating the PI3K/AKT pathway (22). Additionally, DHCR24 has been reported to activate the PI3K/AKT pathway to alleviate dilated cardiomyopathy in mice (31). Furthermore, DHCR24 overexpression has been shown to trigger microglia polarization and inflammation by activating the AKT/GSK3β signaling pathway in BV-2 cells (32). Our research revealed similar results, demonstrating that DHCR24 inhibition significantly inactivated the PI3K/AKT/GSK3β pathway in DDP-resistant NSCLC cells. Moreover, activation of PI3K/AKT counteracted the ferroptosis and proliferation inhibition induced by DHCR24 silencing in NSCLC cells upon DDP treatment. Therefore, we concluded that DHCR24 knockdown triggers ferroptosis in NSCLC cells via inactivation of the PI3K/AKT/GSK3β pathway.

This study has several limitations. First, the clinical sample size is small, and the correlation between abnormal expression of DHCR24 and DDP resistance needs to be validated in a larger cohort of patients. Second, the upstream regulatory mechanisms responsible for the high expression of DHCR24 during DDP resistance have not been elucidated. Future research should further explore the mechanisms underlying DHCR24 to provide foundational data for its clinical application.

### 5.1. Conclusions

Our observations demonstrated that DHCR24 knockdown enhances DDP sensitivity in NSCLC cells by inactivating the PI3K/AKT/GSK3β pathway, thereby restraining ROS-mediated ferroptosis. These findings provide a strong theoretical basis for developing DHCR24-targeted therapies against DDP-resistant NSCLC.

## Data Availability

The dataset presented in the study is available on request from the corresponding author during submission or after its publication. The data are not publicly available due to privacy protection.
